# Antitumor Effects of Trimethylellagic Acid Isolated From *Sanguisorba officinalis* L. on Colorectal Cancer *via* Angiogenesis Inhibition and Apoptosis Induction

**DOI:** 10.3389/fphar.2019.01646

**Published:** 2020-01-28

**Authors:** Chongfei Bai, Yueshan Sun, Xianchao Pan, Jing Yang, Xiaoxuan Li, Anguo Wu, Dalian Qin, Shousong Cao, Wenjun Zou, Jianming Wu

**Affiliations:** ^1^ Department of Pharmacology, School of Pharmacy, Southwest Medical University, Luzhou, China; ^2^ Department of Chinese Materia Medica, School of Pharmacy, Chengdu University of Traditional Chinese Medicine, Chengdu, China; ^3^ Department of Medicine, School of Pharmacy, Southwest Medical University, Luzhou, China; ^4^ Institute of Cardiovascular Research, Key Laboratory of Medical Electrophysiology, Luzhou Key Laboratory of Activity Screening and Druggability Evaluation for Chinese Materia Medica, Luzhou, China; ^5^ Department of Pharmacy, Affiliated Hospital of Southwest Medical University, Luzhou, China

**Keywords:** 3,3',4'-trimethylellagic acid, antitumor effects, antiangiogenesis, cytotoxicity, apoptosis

## Abstract

Previous studies have demonstrated that tannin could inhibit the proliferation and angiogenesis of cancer cells. However, the mechanism(s) associated with its antitumor effect remains unclear. Here, we investigated the effects of 3,3',4'-trimethylellagic acid (TMEA), a tannin compound isolated from *Sanguisorba officinalis* L., on the proliferation, angiogenesis, and apoptosis in cancer cells, as well as the underlying mechanism(s) related to its antitumor activity. TMEA was isolated from *Sanguisorba officinalis* L. by silica gel column chromatography. Molecular docking was carried out to assess active pocket binding between TMEA and vascular endothelial growth factor receptor 2 (VEGFR2). The antiangiogenic effect of TMEA on the migration and tube formation was detected in HUVECs by wound healing and tube formation assays, respectively. The antitumor effects of TMEA on the cell proliferation were determined in HepG2, A549, and SW620 cells by MTS assay *in vitro* and on the tumor growth of SW620 xenografts bearing in nude mice *in vivo*. The mRNA expression of Bcl-2, Bax, caspase-3, VEGF, PI3K, and mTOR were measured by qRT-PCR and protein expression of Bcl-2, Bax, caspase-3, VEGF, PI3K, and mTOR by Western blotting, and the protein expression of Bcl-2, Bax, caspase-3 and CD31 were detected by immunohistochemical analysis *in vivo*, respectively. The results showed that TMEA combined with VEGFR2 in the functional pockets of Asn223A, Gly922A, and Leu840A and inhibited the proliferation, migration, tube formation, and expression of VEGF and its downstream signaling mediators in HUVECs. TMEA also significantly inhibited the proliferation of HepG2, A549, and SW620 cancer cells *in vitro*, and suppressed the growth of SW620 tumors *in vivo*. Moreover, TMEA upregulated the expression of proapoptotic factors Bax and caspase-3 and downregulated the expression of antiapoptotic factors CD31 and Bcl-2 in cancer cells and/or tumor tissues. The data indicate that TMEA executes its anticancer activity by inducing apoptosis and inhibiting angiogenesis in cancer cells *in vitro* and tumor growth *in vivo*. The underlying anticancer mechanism is associated with the apoptotic and VEGF/PI3K/AKT/mTOR pathways.

## Introduction

Cancer is one of the leading causes of death worldwide. The incidence and mortality rates of cancer have tremendously increased, constituting an enormous burden on public health, cancer patients and their families, and society as a whole (Torre et al., 2016; Gonzaga et al., 2018). Therefore, various strategies in cancer therapy to improve therapeutic efficacy have been developed in preclinical and clinical settings recently. These treatment strategies mainly include novel therapeutic targets, such as antiangiogenesis, apoptosis-related signaling pathways, oncogene inactivation, and tumor suppressors and immunotherapy (Cross and Burmester, 2006). Among these strategies, induction of apoptosis and repression of angiogenesis are considered two promising approaches in cancer therapy (Ding and Fisher, 2002; Carmeliet and Jain, 2011; Potente et al., 2011). For example, sorafenib, a small molecule inhibitor of several kinases including Raf, vascular endothelial growth-factor receptors (VEGFR) and platelet-derived growth factor receptor (PDGFR) involved in the proliferation and angiogenesis of cancer cells, showed significantly therapeutic efficacy in the treatment of patients with various cancers, including liver, lung, and colorectal cancers clinically (Rini, 2006; Blumenschein, 2008; Mellema et al., 2013; Ogasawara et al., 2017).

Angiogenesis plays a crucial role in tumor growth by ensuring the supply of sufficient oxygen and fundamental nutrients to cancer cells and tissues (Tabata, 2003; Zhu et al., 2010). Angiogenesis is regulated by various pro-angiogenic factors such as VEGF, PDGF, basic fibroblast growth factor (bFGF), transforming growth factor-beta (TGF-β), angiogenin, and interleukin-8 (IL-8) ect. These factors promote the stimulation, proliferation, and invasion of endothelial cells, resulting in the excessive growth of new capillaries into the tumor (Verheul et al., 2004; Kerbel, 2008). VEGF is one of the most important regulators of angiogenesis (Shibuya, 2001). VEGF is secreted from cancer and stromal cells in the tumor microenvironment for promoting the growth and migration of vascular endothelial cells and the permeability of blood vessels (Ferrara, 2004). Moreover, several key intracellular signaling molecules including phosphoinositide 3-kinase (PI3K), protein kinase B (AKT), and mammalian target of rapamycin (mTOR) are activated when VEGF binds to VEGFR2 on endothelial cells, which are responsible for the proliferation, migration, invasion, and survival of endothelial cells (Stefanini et al., 2009; Kim et al., 2013; Jiao et al., 2016; Kim, 2017). Therefore, anti-VEGF therapy has clearly demonstrated antitumor efficacy *via* inhibition of angiogenesis against various malignancies clinically (Grothey and Galanis, 2009).

Aberrant apoptosis is a major reason for cancer development, survival, and progression (Lowe and Lin, 2000; Tayyaba et al., 2016). The ability to evade apoptosis is an important feature of cancer cells. Bcl-2 and Bax belong to the Bcl-2 family, which are the most important apoptosis regulatory molecules (Liu et al., 2011; Yao et al., 2017). Bcl-2 and Bax play important roles in the mitochondrial apoptotic pathway, with both factors having opposing functions (Liang et al., 2016). The ratio of Bcl-2 and Bax affects the relative sensitivity or resistance of cancer cells to apoptotic stimuli and therapeutic drugs (Liu et al., 2011). Caspase-3, a downstream effector molecule, is a proteolytic enzyme that executes apoptotic cell death. Therefore, apoptosis is a key target for cancer therapy.

*Sanguisorba officinalis* L. is a traditional Chinese herb that is widely used for immunomodulation and treatment of blood toxicity, hepatitis B, and cancer (Kim et al., 2001; Cai et al., 2012; Wang et al., 2012; Yang et al., 2015; Liu et al., 2016). Tannin, one of the main components of *Sanguisorba officinalis* L., exhibits antibiotic, antiviral, and hematopoietic effects (Sharma et al., 2011; Adini et al., 2017). Recent pharmacological studies have shown that tannin could inhibit the growth of breast cancer cells and angiogenesis of human umbilical vein endothelial cells (HUVECs) (Wang et al., 2012). Moreover, previous study revealed that ellagic acid suppressed angiogenesis in HUVECs and exhibited antitumor activity against sarcoma S180 and liver cancer H22 (Ya et al., 2015). However, the study of the effects of 3,3',4'-trimethylellagic acid (TMEA, an ellagic acid) on the anticancer activity and angiogenesis is limited.

To determine the antitumor effects of TMEA, the cell proliferation was determined by MTS and the mRNA and protein expressions of Bcl-2, Bax, and caspase-3 in liver cancer HepG2, lung cancer A549, and colon cancer SW620 cells by qRT-PCR and Western blotting analysis, respectively. Furthermore, the antitumor activity of TMEA was evaluated in SW620 tumor xenograft bearing in nude mice *in vivo* and the expressions of CD31, Bcl-2, Bax, and caspase-3 were investigated in SW620 tumor tissues by immunohistochemical analysis. In addition, the effects of TMEA on molecular docking with VEGFR2, VEGF expression, and VEGF-induced angiogenesis were investigated by wound healing and tube formation assay in HUVECs.

## Methods

### Cell Culture

The hepatoma cell line HepG2, non-small lung cancer cell line A549, and colon cancer cell line SW620 were purchased from the China Center for Type Culture Collection (CCTCC, Wuhan, Hubei, China). HepG2 cells were cultured in *Dulbecco's Modified Eagle Medium* (DMEM, Gibco, Thermo Fisher Scientific, Waltham, MA, USA), while A549 and SW620 cells in RPMI 1640 (Gibco, Thermo Fisher Scientific, Waltham, MA, USA). Cultures were supplemented with 10% fetal bovine serum (FBS, Gibco, Thermo Fisher Scientific, Waltham, MA, USA), 100 U/ml penicillin, and 100 μg/ml streptomycin (Beyotime, Sichuan, China) at 37°C in a humidified incubator with a 5% CO_2_ atmosphere. HUVECs were purchased from ScienCell (San Diego, California, USA) and maintained in *Extracellular Matrix* (ECM, ScienCell, San Diego, California, USA) containing 5% FBS, 1% Endothelial Cell Growth Supplement (ECGS), 100 U/ml penicillin, and 100 µg/ml streptomycin at 37°C in a 5% CO_2_ atmosphere.

### Preparation of TMEA

TMEA was extracted from the dried roots of *Sanguisorba officinalis* L. purchased from the Chengdu HeHuaChi medicinal materials market (Chengdu, Sichuan, China) in 2015 and identified by Professor Xianming Lu of Chengdu University of Traditional Chinese Medicine (Chengdu, Sichuan, China). The voucher specimen (SWMU-2015101301) was deposited at Herbarium of Traditional Chinese Medicine, School of Pharmacy, Southwest Medical University showed in **Figure S1**. The material (50 kg) was ground into a powder, and 70% ethyl alcohol products were obtained by percolation. The extract was partitioned with methylene chloride, and then the solvent was removed. The CH_2_Cl_2_ fraction was subjected to chromatographic isolation by silica gel and eluted with petroleum ether (PE)-acetic ether (EAC) (8:2), PE-EAC (6:4), PE-EAC (8:2), and PE-EAC (10:0), successively. TMEA (1.2 g) was obtained in PE–EAC (8:2) eluted solution and was confirmed by UHPLC-TOF-MS, ^13^C-NMR, and ^1^H-NMR, respectively. Furthermore, the stability of TMEA *in vitro* was performed by detecting the content of 30 μM TMEA in DMSO within 7 days using ultra high performance liquid chromatography (UHPLC) equipped with a diode-array detector. The mobile phase composed of (A) acetonitrile and (B) 0.1% formic acid in water was set as follow: 0.01–10.0 min (60% B), 10.01–15.00 min (60% B–20% B), 15.01–16.00 min (20% B–0% B), 16.01–17.00 min (0% B–60% B), and 17.01–21.00 min (60% B) at a flow rate of 1 ml/min.

### Molecular Docking

The X-ray crystal structure of VEGFR2 in complex with axitinib was retrieved from the Protein Data Bank (PDB code: 4AGC). The crystallized waters were removed, and hydrogen atoms were added. TMEA was optimized with the MMFF94 force field. Molecular docking was performed by the fully automatic flexible Surflex-Dock (built-in Sybyl 8.1) method as described previously (Costache et al., 2015). The conformation search in Surflex-Dock was guided by a “protomol”. Cocrystallized axitinib was used to generate the protomol using a thresh of 0.5 and a bloat of 3. The docking poses of TMEA were sorted by total scores expressed in -log_10_ (Kd) units, consisting of hydrophobic, polar, electrostatic, repulsive, entropic, solvation, and crash terms. Both number of starting conformations and retained docking poses were set to 10. Other docking parameters were set by default.

### Cell Viability Assays by MTS

Cell proliferation was evaluated by MTS (Promega, Madison, WI, USA, G5421). HepG2, A549, and SW620 cells were seeded in 96-well plates at the densities of 5 × 10^3^ cells/well for HepG2 and A549, 1 × 10^4^ cells/well for SW620, and 3 × 10^3^ cells/well for HUVECs. The cells were treated with various concentrations of TMEA (3, 15, 30, and 60 μM) or vehicle solution (control) for 24 h, 36 h, 48 h, or 60 h, respectively. Then, the treated cells were incubated with culture medium containing 10% MTS reagent for 2 h, and the absorbance was recorded at 490 nm using a Thermo Scientific Microplate Reader. The relative inhibition rate of cell growth was calculated according to the formula: $R=\left[\frac{\text{A}2-\text{A}1}{\text{A}2}\right]\times 100\%$ as previously described (Wei et al., 2012). A1 is the mean absorbance value of drug treated cells and A2 is the mean absorbance value of control cells treated with vehicle solution. All experiments were performed at least three times in triplicate.

### Wound Healing

The effect of TMEA on the migration of HUVECs was evaluated as previously described (Walter et al., 2015). After HUVECs were scratched with a sterile pipette tip, the cells were treated with medium containing 0, 3, 15, 30, and 60 μM of TMEA and 10 μM of sorafenib (TargetMol, Boston, MA, USA) for 24 h. Three randomly selected views along the scraped lines were photographed on each well at 0 and 24 h after TMEA treatment under a phase-contrast inverted microscope at a magnification of 40×. The horizontal distance was quantified at 0 and 24 h for the migration ability using Image-Pro Plus software 6.0 (Media Cybernetics, Inc., Rockville, MD, USA). The migration distance was calculated as follows: migration distance = distance within scratch (0 h) − distance within scratch (24 h).

### Tube Formation Assay

Chilled liquid Matrigel (Corning Inc, Corning, NY, USA) was dispensed into 96-well plates (50 μl per well) and allowed to solidify for 30 min at 37°C. HUVECs (4 × 10^4^ cells) were maintained in 100 μl complete medium and treated with the indicated concentrations of TMEA in Matrigel-coated plates at 37°C in a humidified 5% CO_2_ atmosphere for 6 h. The images of tubular structures were captured with a computer-assisted inverted microscope at 40× magnification. The enclosed networks of complete tubes were photographed and counted from three randomly chosen fields.

### Establishment of SW620 Tumor Xenografts in Nude Mice and Drug Administration *In Vivo*

Forty 6-week-old male Balb/c nude mice (body weight: 16–20 g) were purchased from the Chengdu Dossy Animal Experimental Company (Chengdu, Sichuan, China, license No. SCXK 2013-24). All mice had free access to food and water and were housed up to five mice per cage under constant conditions with temperatures of 22 to 25°C, a relative humidity of 55%, and a 12-h light/dark cycle. The experimental protocol animal ethics were approved (Permit No. 20180329) by the Committee on Use and Care of Animals of Southwest Medical University (Luzhou, Sichuan, China).

SW620 tumor xenografts were established by hypodermic injection of SW620 cells (1 × 10^7^ cells/ml in 100 μl of sterile PBS) in the dorsal aspect of the right thigh of mice. The tumor-bearing mice were randomly divided into five groups on days 14 after cells injection when the tumors reached to about 100 mm^3^ (mg): 1). control; 2). 5-FU (Tianjin jinyao pharmaceutical co. LTD, Tianjing, China), 25 mg/kg; 3). TMEA 50 mg/kg; 4). TMEA 100 mg/kg; and 5). TMEA 200 mg/kg. TMEA was prepared by addition of carboxymethylcellulose (CMC, 1%), Tween-80 (1%), and polyethylene glycol 400 (PEG400, 1%) in normal saline (Choi et al., 2015) and orally (p.o.) administered to mice once a day (daily) for 3 weeks and control mice were treated with an equal volume of solvent (vehicle) at the same schedule and duration of TMEA. 5-FU was prepared with normal saline and given as intraperitoneal injection (i.p.) every 2 days for 3 weeks. There are eight mice for each group. All animals were observed daily for toxicity and tumor volumes were measured and recorded for two perpendicular diameters (a and b) of the tumor every 3 days. The weight of the tumor tissue was measured and the tumor volume (V) was estimated using the formula as V = ab^2^/2, in which “a” is the longest axis and “b” is the shortest axis of the tumor. All mice were euthanized by cervical dislocation at the end of experiment and parts of the tumor tissues were fixed in 4% paraformaldehyde and subsequently dehydrated and embedded in paraffin for immunohistochemical staining.

### Immunohistochemistry

The tumor tissues were deparaffinized in xylene and rehydrated through graded alcohol followed by immunohistochemical staining. Then, the samples were paraffin-embedded and sectioned (5 μm) as previously described (Wang et al., 2012). Sections were subjected to deparaffinization and antigen retrieval, followed by a brief treatment with 3% H_2_O_2_ for 10 min to neutralize endogenous peroxidase activity. Hydrated paraffin sections were incubated in blocking solution for 30 min at room temperature and then at 4°C overnight with CD31 (1:25), Bcl-2 (1:250), Bax (1:250), (Cell Signaling Technology, Danvers, MA, USA), and caspase-3 (1:100, Abcam, Cambridge, UK), respectively; and detected with avidin-biotin-HRP complex and DAB as the chromogen. Nuclei were counterstained with hematoxylin. High-quality images were captured in six fields per tumor sample with CCD (DP73; Olympus, Tokyo, Japan). Semiquantitative analysis was measured by determining the integral and average optical densities.

### qRT-PCR Analysis

SW620 cells were seeded into 6-well plates at a density of 2 × 10^6^ cells/well. After 5 h of incubation for attachment, the cells were cultured in various concentrations of TMEA (0, 3, 15, 30, and 60 μM) and sorafenib (10 μM) for 12 h. Total RNA was extracted using TRIzol reagent. Reverse transcription was performed using a First Strand cDNA synthesis kit (TAKARA, Tiantai life technology co. LTD, Chengdu, Sichuan, China) according to the manufacturer's instructions. The obtained cDNA was used to assess the mRNA of Bcl-2, Bax, and caspase-3 in SW620 cells by PCR with Taq DNA polymerase (Invitrogen, Shanghai, China) using the following primers:

Bcl-2, 5'-CATGTGTGTGGAGAGCGTCA-3' (forward)

5'- CCACGGCACAGAATCCAAAT-3' (reverse)

Bax, 5'-ACAGGGTACGATAACCGGGA-3' (forward)

5'-TCCACGGCACAGAATCCAAA-3' (reverse)

caspase-3, 5'-ATCTCGGTCTGGTACAGATG-3' (forward)

5'-TCAACACCACTGTCTGTCTC-3' (reverse)

GAPDH, 5'-CCTCCTGTTCGACAGTCAGC-3' (forward)

5'- CCTAGCCTCCCGGGTTTCTC-3' (reverse)

HUVECs (1 × 10^5^ cells/well) were seeded into 6-well plates and treated with 10 μM sorafenib and various concentrations of TMEA (0, 3, 15, 30, and 60 μM) for 36 h. The primers for VEGF, PI3K, mTOR, and GAPDH were designed as follows:

VEGF, 5'-AAATGGTTTCACGCCACACG-3' (forward)

5'-CAGTGCCAAGTTCCAAAGGC-3' (reverse)

PI3K, 5'-AAATGGTTTCACGCCACACG-3' (forward)

5'-CAGTGCCAAGTTCCAAAGGC-3' (reverse)

mTOR, 5'-CTTAGAGGACAGCGGGGAAG-3' (forward)

5'-TCCAAGCATCTTGCCCTGAG-3' (reverse)

GAPDH, 5'-CCTCCTGTTCGACAGTCAGC-3' (forward)

5'-CCTAGCCTCCCGGGTTTCTC-3' (reverse)

GAPDH was used as a loading control. The level of mRNA expression was calculated using the 2^−(ΔΔCT)^ method.

### Western Blotting Analysis

Cells (1 × 10^5^) were seeded into 6-well plates. After 12 h of incubation, the cells were treated with vehicle (control), 5-FU (40 μM), or various concentrations of TMEA (0, 3, 15, 30, and 60 μM) and incubated at 37°C under a 5% CO_2_ atmosphere for 48 h. Proteins were extracted according to standard procedures as described previously (Wu et al., 2017) and separated on SDS-PAGE under reducing condition. The membranes were incubated with the following primary antibodies: anti-VEGF (Abcam, Cambridge, UK), anti-PI3K, anti-mTOR, anti-AKT, anti-phospho-PI3K, anti-phospho-mTOR, and anti-phospho-AKT (Cell Signaling Technology, Danvers, MA, USA) at 1:1,000 in HUVECs; anti-Bcl-2 (1:500), anti-Bax (1:600), and anti-caspase-3 (1:300) in SW620 cells and developed with ECL reagent (4A Biotech Co., Ltd, Beijing, China). The protein band intensity was quantified using Image J (National Institutes of Health, Bethesda, MD, USA). The ratio of the targeted proteins to β-actin was calculated. β-actin was used as a loading control.

### Statistical Analysis

All the data were reported as the mean ± standard deviation (SD). Difference between groups was analyzed by one-way univariate analysis of variance (ANOVA). A *p-*value less than 0.05 (*p* < 0.05) was considered to be statistically significant (marked as *). Higher significance levels were established at *p* < 0.01 (marked as **).

## Results

### Preparation of TMEA From *Sanguisorba officinalis* L.

**Figure 1:**
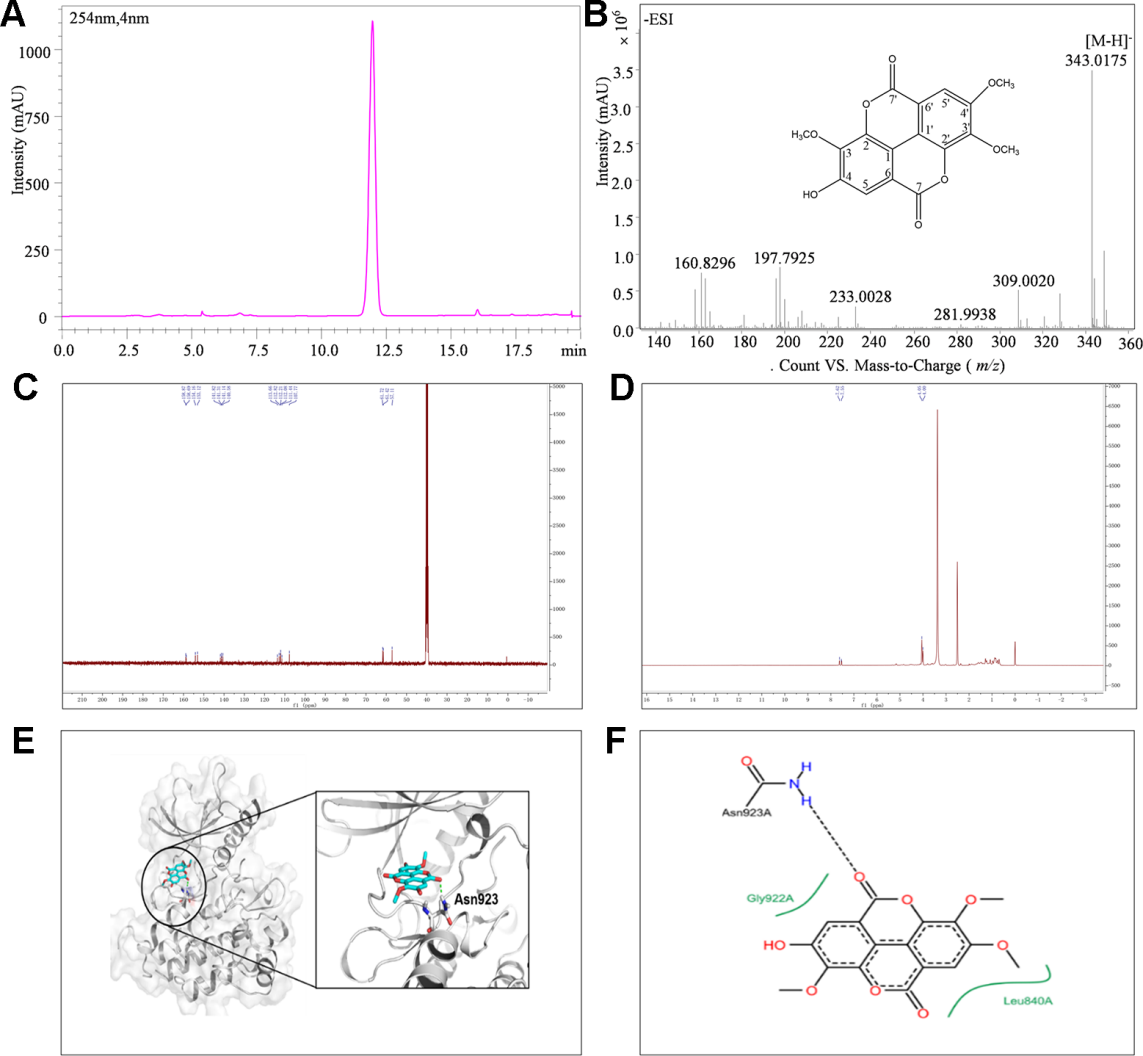
The structure and purity of TMEA. **(A)** Determination of TMEA by HPLC chromatography; **(B)** The molecular formula and determination of TMEA by MS chromatography; **(C)** Determination of TMEA by ^13^C-NMR chromatography; **(D)** Determination of TMEA by ^1^H-NMR chromatography; **(E)** The best scored docking pose of TMEA in the binding pocket of VEGFR2; **(F)** The interactions between TMEA and VEGFR2. TMEA, 3,3',4'-trimethylellagic acid; VEGFR2, vascular endothelial growth factor receptor 2.

The isolated CH_2_Cl_2_ fraction from *Sanguisorba officinalis* L. was determined as 3,3', 4'-trimethoxy ellagic acid (TMEA). TMEA solution (30 μM) in pure DMSO was a homogeneous phase system without any crystals. The composition of TMEA was confirmed by UHPLC-TOF-MS, ^13^C-NMR, and ^1^H-NMR, respectively. As shown in **Figure 1B**, the parameter of ESI-MS *m/z* was identified as 343.0[M-H]^-^. The parameters of ^13^C-NMR chromatography (**Figure 1C**) were identified as δ: 111.44 (C-1), 141.31 (C-2), 140.58 (C-3), 153.12 (C-4), 112.08 (C-5), 112.82 (C-6), 158.68 (C-7), 112.21 (C-1'), 141.82 (C-2'), 141.13 (C- 3'), 154.16 (C-4'), 107.77 (C-5'), 113.66 (C-6'), 158.86 (C-7'), 57.10 (OMe-4'), 61.72 (OMe-3'), and 61.42 (OMe-3). The parameters of ^1^H-NMR chromatography (**Figure 1D**) were δ: 7.53 (^1^H singlet), 7.62 (^1^H singlet), 4.00 (^1^H singlet), 4.04 (^1^H singlet), and 4.05 (^1^H singlet), respectively. These results were consistent with those previously reported and identified as TMEA (Khac et al., 1990). The purity of TMEA was 98.35% determined by UHPLC (**Figure 1A**). In addition, the peak area of TMEA between the 0^th^ and the 7^th^ day had no significant change (**Figure S2**), suggested that TMEA was stable at least within 7 days.

### Molecular Docking

The binding mode of TMEA in the drug-binding pocket of VEGFR2 was predicted by the Surflex-dock method (**Figures 1E, F**). TMEA binds to VEGFR2 through a H-bond with Asn923 and hydrophobic interactions with Leu840 and Gly922, resulting in a relatively stable complex.

### TMEA Inhibits the Proliferation of Cancer Cells

**Figure 2:**
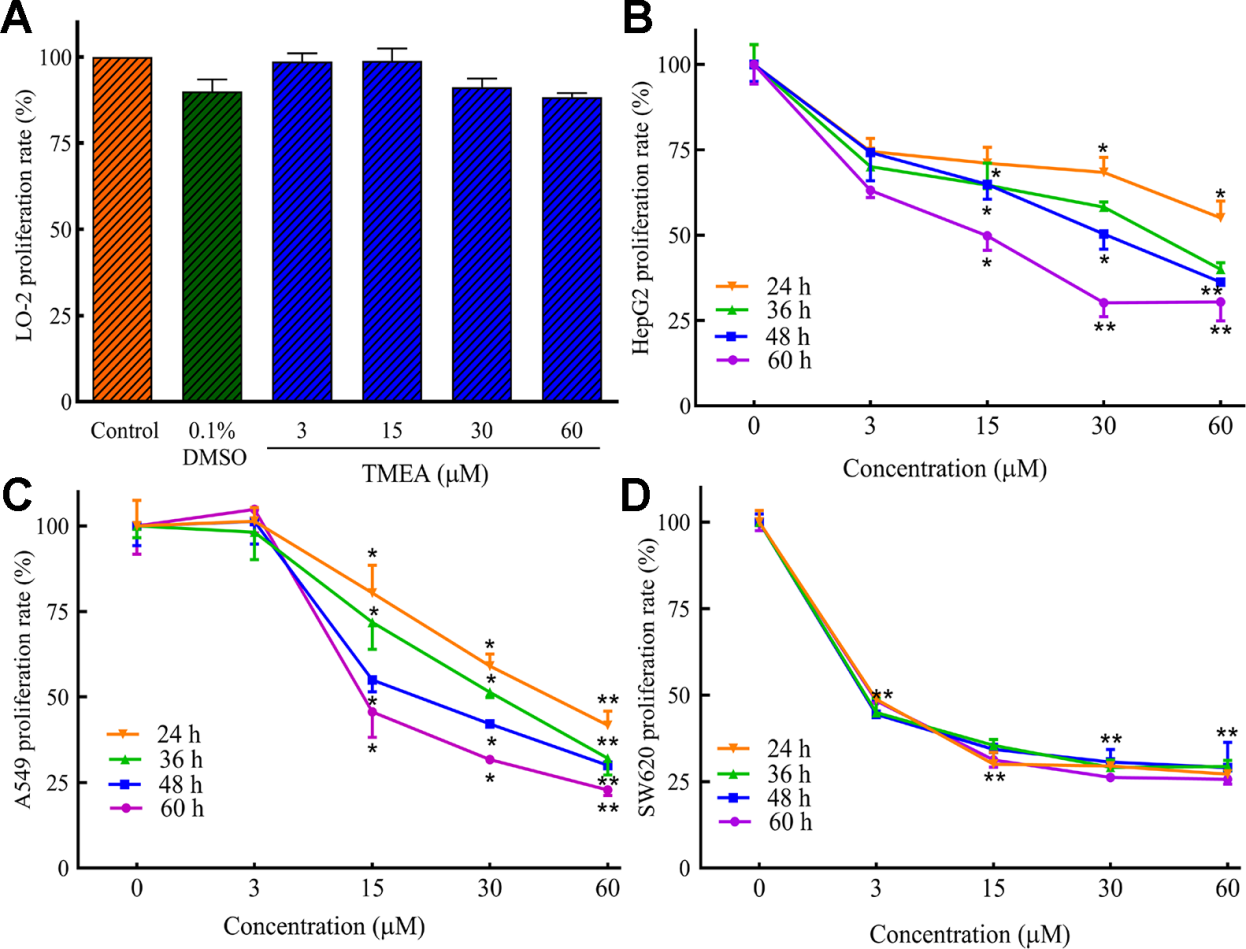
Inhibitory effect of TMEA on the proliferation rates of LO-2 **(A)**, HepG2 **(B)**, A549 **(C)**, and SW620 **(D)** cells by MTS analysis. The data are presented from at least three independent experiments run in triplicate and expressed as mean ± SD. *p < 0.05, **p < 0.01 compared to the vehicle-treated controls by one-way univariate analysis of variance (ANOVA). TMEA, 3,3',4'-trimethylellagic acid.

Before the inhibition test for the cancer cells, we evaluated the cytotoxicity of TMEA against normal hepatic LO-2 cells to explore its safe concentration. Then, we evaluated the potential inhibitory effect of TMEA on the proliferation of multiple human cancer cells. HepG2, A549, and SW620 cells were treated with various concentrations of TMEA (3, 15, 30, and 60 μM) or vehicle solution (control) for 24, 36, 48, or 60 h and the cell viabilities were determined by MTS assay. The results showed that TMEA at the concentration of 60 μM was acceptable concentration against LO-2 cells because it had only limited cytotoxicity without significant difference compared to that of vehicle control (**Figure 2A**). However, TMEA significantly inhibited the growth of HepG2, A549, and SW620 (**Figures 2B–D**) cells compared to that of control cells treated with vehicle. The results indicated that TMEA exhibited high selection against cancer cells by effectively inhibiting the proliferation of HepG2, A549, and SW620 cancer cells but not the hepatic LO-2 normal cells.

### TMEA Suppresses Angiogenesis in HUVECs

**Figure 3:**
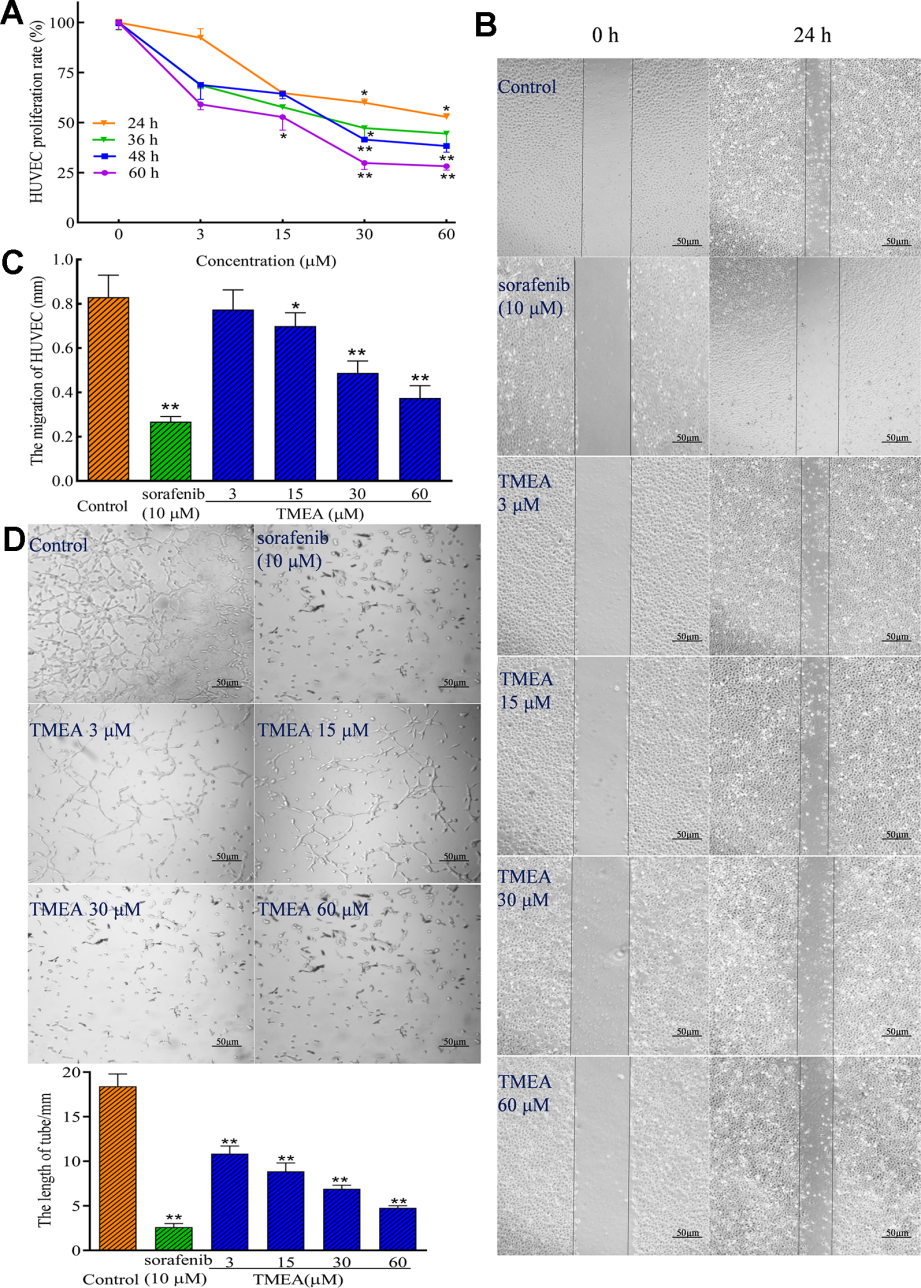
Inhibitory effect of TMEA on the growth and migration of HUVECs. **(A)** The proliferation rate of HUVECs were determined by the MTS assay; **(B)** The migration pattern of HUVECs after treatment of various concentrations of TMEA (3, 15, 30, and 60 μM) or sorafenib (10 μM) at 0 and 24 h under a phase-contrast microscopy; **(C)** Summary bar graph of the effect of TMEA and sorafenib on migration of HUVECs treated with TMEA or sorafenib for 24 h. **(D)** Effect of TMEA and sorafenib on tube formation of HUVECs. The data are presented from at least three independent experiments run in triplicate and expressed as mean ± SD. *p < 0.05, **p < 0.01 compared with the vehicle-treated controls by one-way univariate analysis of variance (ANOVA). TMEA, 3,3',4'-trimethylellagic acid.

To investigate the effect of TMEA on angiogenesis, the proliferation, migration, and tube formation of HUVECs were determined by MTS, wound healing, and Matrigel-based tube formation assays (**Figures 3A–D**), respectively. The results demonstrated that TMEA at the concentrations of 15, 30, and 60 μM significantly inhibited (*p* < 0.05 or *p* < 0.01) the proliferation and migration of HUVECs (**Figures 3A–C**). Tubule formation or tube-like structures can be measured *in vitro* because endothelial cells differentiate and rapidly form tube-like structures when cultured on matrix basement membranes (Staton et al., 2009). Therefore, we also investigated the effect of TMEA on the tube formation of HUVECs and the results showed that TMEA at the concentrations of 3, 15, 30, and 60 μM significantly inhibited (*p* < 0.01) the tube formation of HUVECs (**Figure 3D**). Sorafenib (a commonly clinically used anticancer drug) at 10 μM also significantly inhibited (*p* < 0.01) the proliferation, migration and tube formation in HUVECs. The data suggested that TMEA effectively inhibited the proliferation, migration, and tube formation of HUVECs.

### TMEA Inhibits the Growth of SW620 Tumor Xenografts Bearing in Nude Mice *In Vivo*

**Figure 4:**
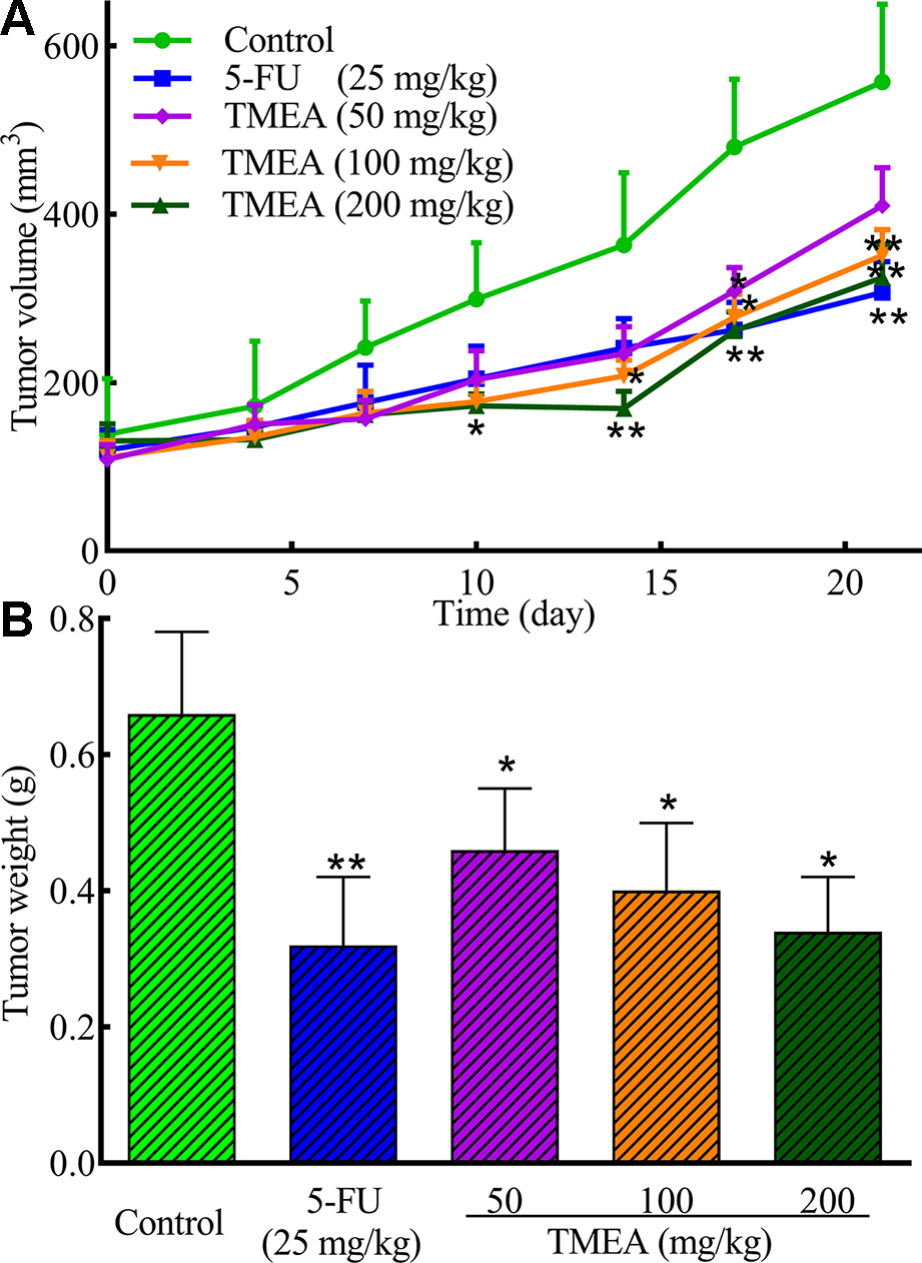
TMEA inhibits the tumor growth of SW620 xenograft bearing in nude mice. **(A)** Tumor volume and **(B)** Tumor weight of the SW620 xenografts bearing in nude mice after treatment of vehicle solution (control), 5-FU (25 mg/kg, i.p. twice a week for 3 weeks) or TMEA (50, 100, and 100 mg/kg, p.o., daily for 3 weeks). There were eight mice for each group and the data are expressed as mean ± SD. *p < 0.05, **p < 0.01, compared with the control by one-way univariate analysis of variance (ANOVA). TMEA, 3,3',4'-trimethylellagic acid.

The antitumor effect of TMEA was further evaluated in SW620 tumor xenografts bearing in nude mice *in vivo*. Daily oral administration of TMEA at 50–200 mg/kg for 3 weeks significantly inhibited (*p* < 0.05) the tumor growth of SW620 tumor xenografts with much less tumor volume and weight compared to that of control (**Figures 4A, B**). Furthermore, TMEA at the dose of 200 mg/kg had similar antitumor efficacy than that of 5-FU (one of the most commonly used chemotherapeutic drug for the treatment of colorectal cancer clinically) at 25 mg/kg (at or near the maximal tolerated dose). The result indicates that TMEA is effective against SW620 tumor xenografts *in vivo*.

### Effects of TMEA on the Expression of CD31, Bcl-2, Bax, and Caspase-3 in the Tissues of SW620 Xenografts

**Figure 5:**
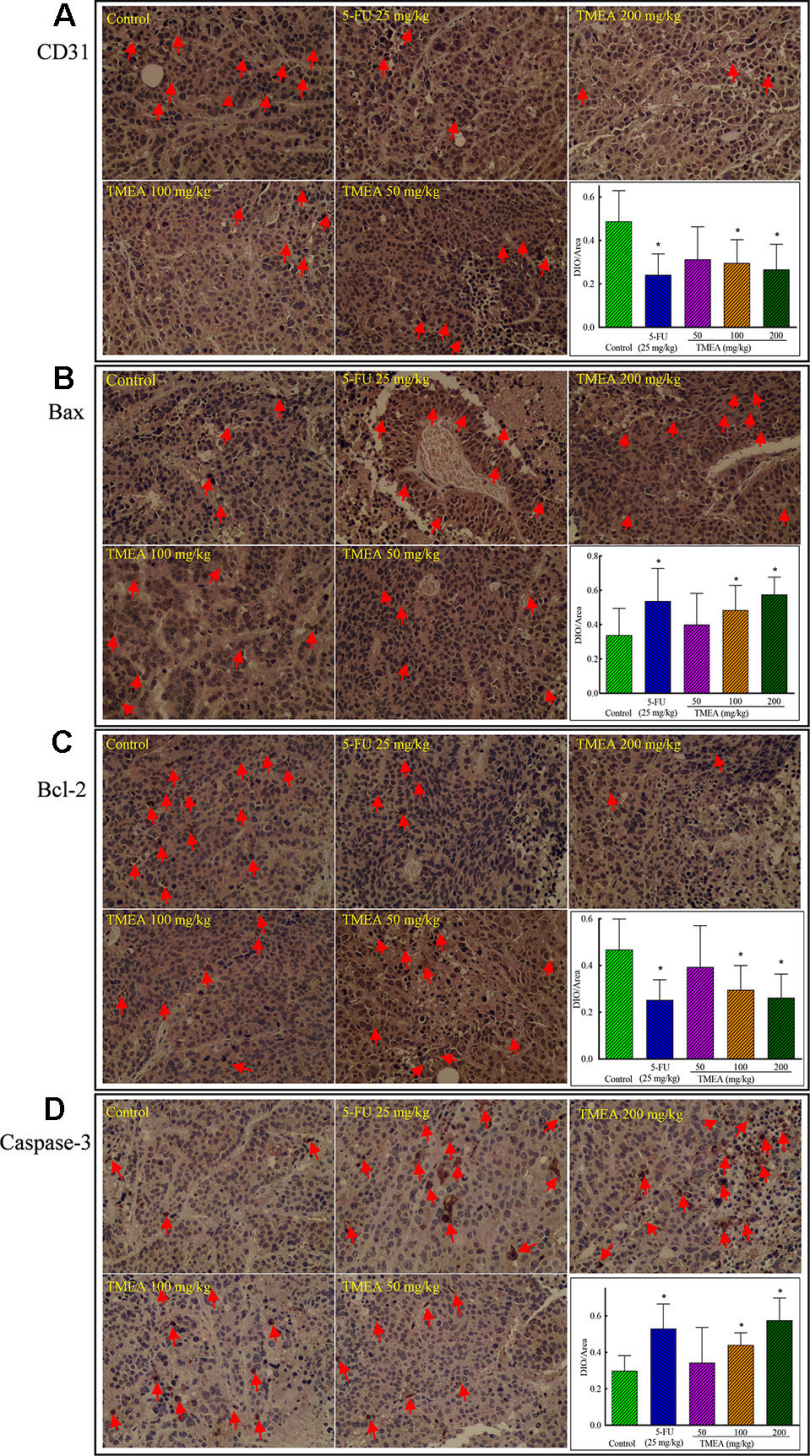
Effect of TMEA and 5-FU on the expression of CD31 **(A)**, Bax **(B)**, Bcl-2 **(C)**, and caspase-3 **(D)** in the tissues of SW620 xenografts *in vivo* (200×). The tumor tissues were removed after tumor-bearing nude mice were treated with vehicle solution (control), 5-FU (25 mg/kg, i.p., twice a week for 3 weeks) or TMEA (50, 100, and 200 mg/kg, p.o., daily for 3 weeks) for 3 weeks. The data are presented from at least three independent samples run in triplicate and expressed as mean ± SD (photographs only show representative data). *p < 0.05, compared with the control by one-way univariate analysis of variance (ANOVA). TMEA, 3,3',4'-trimethylellagic acid.

To investigate the underlying antitumor mechanism of TMEA against SW620 tumor xenografts *in vivo*, we investigated the effects of TMEA on the expression of CD31, Bcl-2, Bax, and caspase-3 and compared to that of 5-FU treatment. The results showed that TMEA at 100 and 200 mg/kg and 5-FU at 25 mg/kg significantly inhibited (*p* < 0.05) the expression of CD31 (**Figure 5A**) and Bcl-2 (**Figure 5C**) compared to that of the control. Moreover, TMEA at 100 and 200 mg/kg and 5-FU at 25 mg/kg also significantly promoted (*p* < 0.05) the expression of Bax (**Figure 5B**) and caspase-3 (**Figure 5D**) in the tumor tissues. TMEA at 50 mg/kg also could downregulate the expression of CD31 and Bcl-2 while upregulate the expression of Bax and caspase-3 without statistically significant different (*p >* 0.05, **Figures 5A–C**). The results indicated that TMEA could significantly downregulate the expression of the antiapoptotic factors CD31 and Bcl-2 and upregulate the expression of proapoptotic factors Bax and caspase-3 in a dose-dependent manner in SW620 tumor tissues.

### Effects of TMEA on the mRNA Expression of Bcl-2, Bax, and Caspase-3 in SW620 Cells and VEGF, PI3K, and mTOR in HUVECs

**Figure 6:**
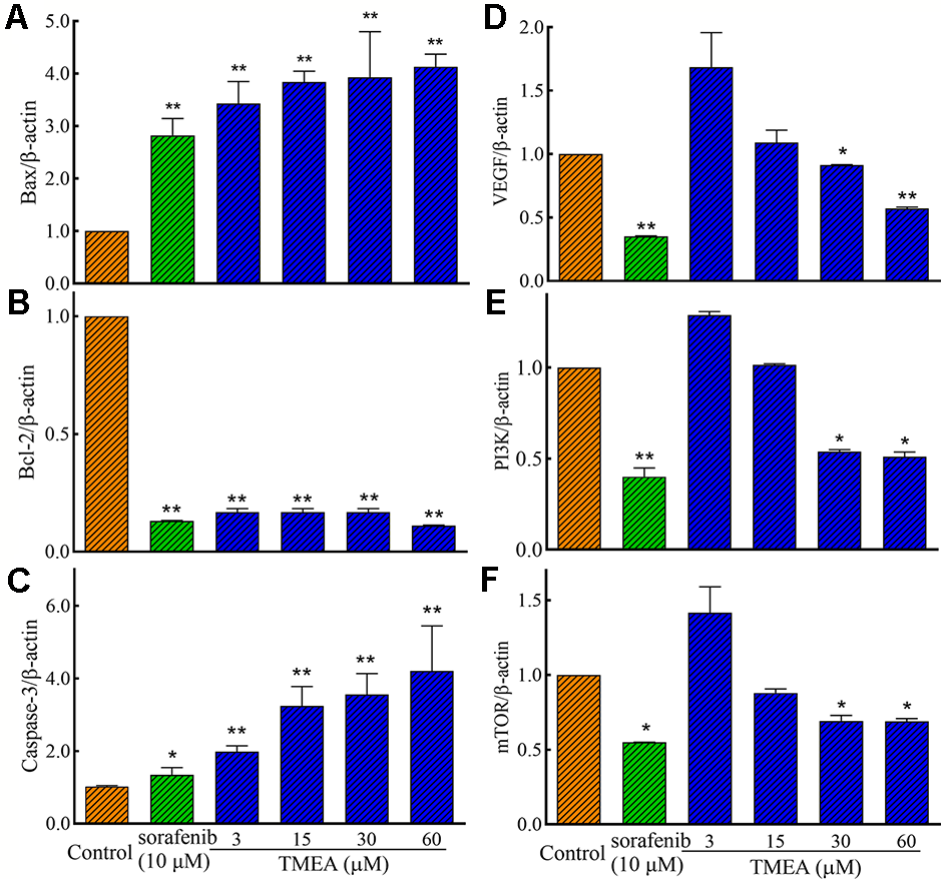
The relative expression of the related mRNA of Bax **(A)**, Bcl-2 **(B)**, caspase-3 **(C)**, in SW620 cells and VEGF **(D)**, PI3K **(E)**, and mTOR **(F)** in HUVECs. The cells were treated with various concentrations of TMEA (3, 15, 30, and 60 μM) or sorafenib (10 μM) for 12 h. The data are presented from at least three independent experiments run in triplicate and expressed as mean ± SD. *p < 0.05, **p < 0.01, compared with the vehicle-treated controls by one-way univariate analysis of variance (ANOVA). PI3K, phosphoinositide 3-kinase.

To investigate the underlying mechanism(s) associated with the antitumor and antiangiogenic activities of TMEA, we assessed the mRNA expression of Bax, Bcl-2, and caspase-3 in SW620 cells and VEGF, PI3K, and mTOR in HUVECs by qRT-PCR analysis. The results showed that TMEA and sorafenib significantly downregulated (*p* < 0.01) the mRNA expression of Bcl-2 and upregulated the mRNA expression of Bax and caspase-3 in SW620 cells (**Figures 6A–C**). Furthermore, TMEA and sorafenib also significantly inhibited (*p* < 0.05 and < 0.01) the mRNA expression of VEGF, PI3K, and mTOR in a concentration-dependent manner (**Figures 6D–F**). The data showed that TMEA had more potent effect than that of sorafenib.

### Effects of TMEA on the Protein Expression of Bcl-2, Bax, and Caspase-3 in SW620 Cells and VEGF, PI3K, AKT, and mTOR in HUVECs

**Figure 7:**
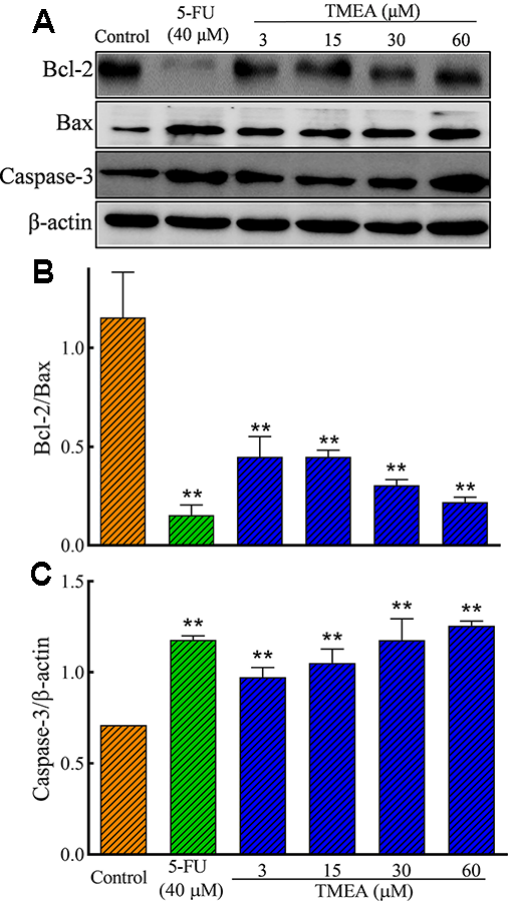
Effect of TMEA and 5-FU on the protein expression of Bcl-2, Bax, and capsase-3 in SW620 cells by Western blot analysis. **(A)** The bands of Bcl-2, Bax, and capsase-3 by Western blotting analysis. β-actin was used as an internal control. **(B)** The ratio of Bcl-2/Bax. **(C)** capsase-3 expression. The cells were treated with vehicle solution (control), 5-FU (40 μM), or various concentrations of TMEA (3, 15, 30, and 60 μM) for 12 h. The data are presented from at least three independent experiments run in triplicate and expressed as mean ± SD. **p < 0.01, compared with the vehicle-treated controls by one-way univariate analysis of variance. TMEA, 3,3',4'-trimethylellagic acid.

We further evaluated the effect of TMEA on the protein expression of Bcl-2, Bax, and caspase-3 in SW620 cells as well as VEGF, PI3K, AKT, and mTOR in HUVECs by Western blotting analysis. As shown in **Figures 7A–C**, TMEA and 5-FU significantly inhibited (*p* < 0.01) the protein expression of Bcl-2 and increased the protein expression of Bax and caspase-3 in a concentration dependent manner.

**Figure 8:**
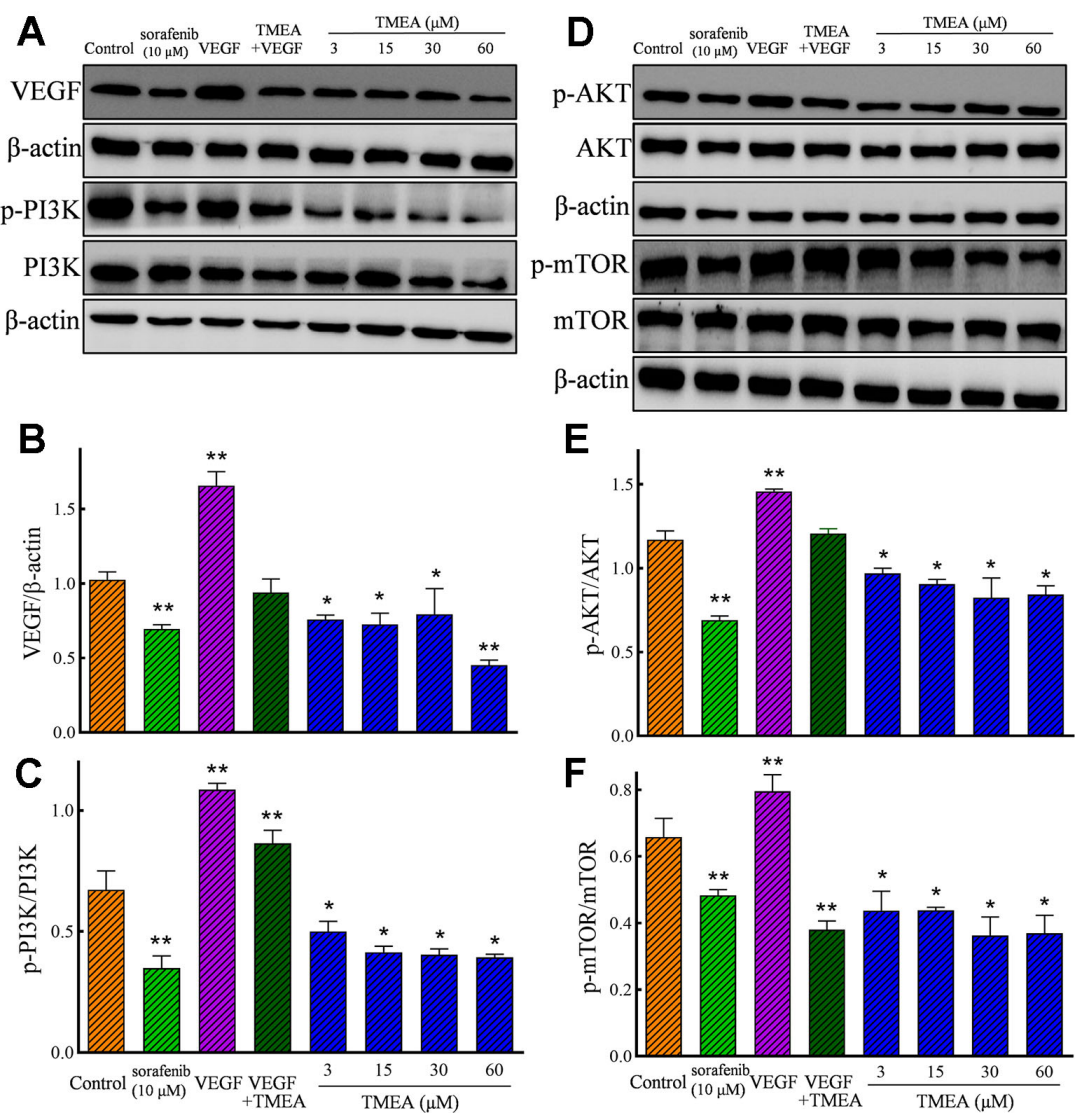
Effect of TMEA on the protein expression and phosphorylation of VEGF downstream signaling molecules, including phosphatidylinositol 3-kinase (PI3K), protein kinase B (AKT) kinase, and mammalian target of rapamycin (mTOR) in HUVECs by Western blotting analysis. **(A)** The bands of VEGF, PI3K, p-PI3K by Western blotting analysis. **(B)** Summary bar graph of VEGF and **(C)** PI3K, p-PI3K. **(D)** The bands of AKT, p-AKT, mTOR and p-mTOR by Western blotting analysis; **(E)** Summary bar graph of AKT, p-AKT, and **(F)** mTOR, p-mTOR. The cells were treated with vehicle solution (control), or sorafenib (10 μM) or various concentrations of TMEA (3, 15, 30, and 60 μM) for 12 h. β-actin was used as an internal control. The data are presented from at least three independent experiments run in triplicate and expressed as mean ± SD. *p < 0.05, **p < 0.01, compared with the vehicle-treated controls by one-way univariate analysis of variance. TMEA, 3,3',4'-trimethylellagic acid; VEGF, vascular endothelial growth factor.

In addition, TMEA and 5-FU also significantly downregulated (*p* < 0.05 or < 0.01) the protein expression of VEGF p-p85 (Tyr458)/PI3K, p-AKT (Ser473)/total AKT, and p-mTOR (Ser2448)/total mTOR in HUVECs (**Figures 8A, D**). However, the total levels of AKT and mTOR were not significantly altered by TMEA treatment. The results suggested that TMEA could inhibit VEGF-stimulated phosphorylation of PI3K, AKT, and mTOR (p-PI3K, p-AKT, and p-mTOR), but not the total levels of AKT and mTOR (**Figures 8A–F**).

## Discussion

Cancer is the primary disease responsible for death worldwide. Despite recent progress and advances in novel antitumor therapeutics such as targeted therapy and immunotherapy, chemotherapy remains the most commonly used treatment modality for patients with cancer. Apoptosis and antiangiogenesis are the key targets for chemotherapeutic drugs. However, drug-induced toxicities and resistance are still the major problems in cancer therapy. The discovery of novel chemotherapeutic drugs from natural compounds provides promising resources (Huang et al., 2019). Tannins are natural compounds that are well-known for their anti-inflammatory and antitumor activities (Kassim et al., 2010; Yuuki et al., 2017). TMEA is isolated and extracted from *Sanguisorba officinalis* L. However, to our best knowledge, the information regarding the antitumor and antiangiogenic activities of TMEA has not been reported. Therefore, in the present study, we evaluated the growth inhibitory activity of TMEA against three different human cancer cells, including HepG2, A549, and SW620 *in vitro* and tumor xenografts of SW620 *in vivo*. Our results demonstrated that TMEA exhibited significant antitumor effects *in vitro* and *in vivo*.

Angiogenesis refers as the formation of new blood vessels from pre-existing ones and is the key factor for the tumor growth and metabolism. VEGF is the prime regulator of pathological angiogenesis (Sharma et al., 2011; Costache et al., 2015; Ya et al., 2015; Adini et al., 2017). HUVECs are ideal cells to investigate angiogenesis *in vitro.* Therefore, we also evaluated the effects of TMEA on the inhibition of the cell growth, migration, and tube formation in HUVECs. Our data revealed that TMEA at the concentration as low as 3 μM significantly inhibited VEGF secretion *in vitro* (**Figure 8A**). In addition, TMEA at 100 and 200 mg/kg markedly decreased CD31 expression in colon cancer SW620 tumor xenografts *in vivo* (**Figure 5**). Moreover, the PI3K/AKT pathway is implicated in multiple cellular processes including cell proliferation, migration, adhesion, and survival. PI3K/AKT pathway is a significant regulator of new blood vessel formation and inhibition of PI3K/AKT/mTOR pathway can suppress angiogenesis by decreasing VEGF expression (Shen et al., 2012). The present findings demonstrated that TMEA downregulated the mRNA expression levels of VEGF, PI3K, and mTOR (**Figures 6D–F**) and inhibited the phosphorylation of PI3K/AKT/mTOR (**Figures 8A–F**). Taken together, the antiangiogenesis effect of TMEA may be mediated *via* VEGF/PI3K/AKT/mTOR signaling pathway to further inhibit tumor growth.

In generally, it was recommended that the dose range of 100–200 mg/kg for *in vivo* studies of extracts (p.o. with the upper limit being much lower for i.p. and i.m. applications), also the 100–200 μg/ml should be assumed as being the upper limit for meaningful pharmacological studies. However, a much lower dose range should be considered for the pure compounds was recommended, (ca. 50 mg/kg for *in vivo* studies of extracts (p.o.), and of 30–50 μM for *in vitro* studies). The use of higher doses needs to be justified in detail (Michael et al., 2020). In the present study, we have performed preliminary experiments and found that the concentration of 60 μM is acceptable because there is no significant difference of cytotoxicity compared to that of vehicle control (medium containing 0.1% DMSO) in the normal hepatic LO-2 cells (**Figure 2A**). As we known, most of the extracts or monomers of botanical drugs have low solubility and bioavailability and the water solubility of TMEA is also not very good so we chose a larger range of concentrations/doses of TMEA to investigate its anticancer activity, which may be a little over the upper limit by the guideline of *Best Practice in Research Overcoming Common Challenges in Phytopharmacological Research* (Michael et al., 2020).

**Figure 9:**
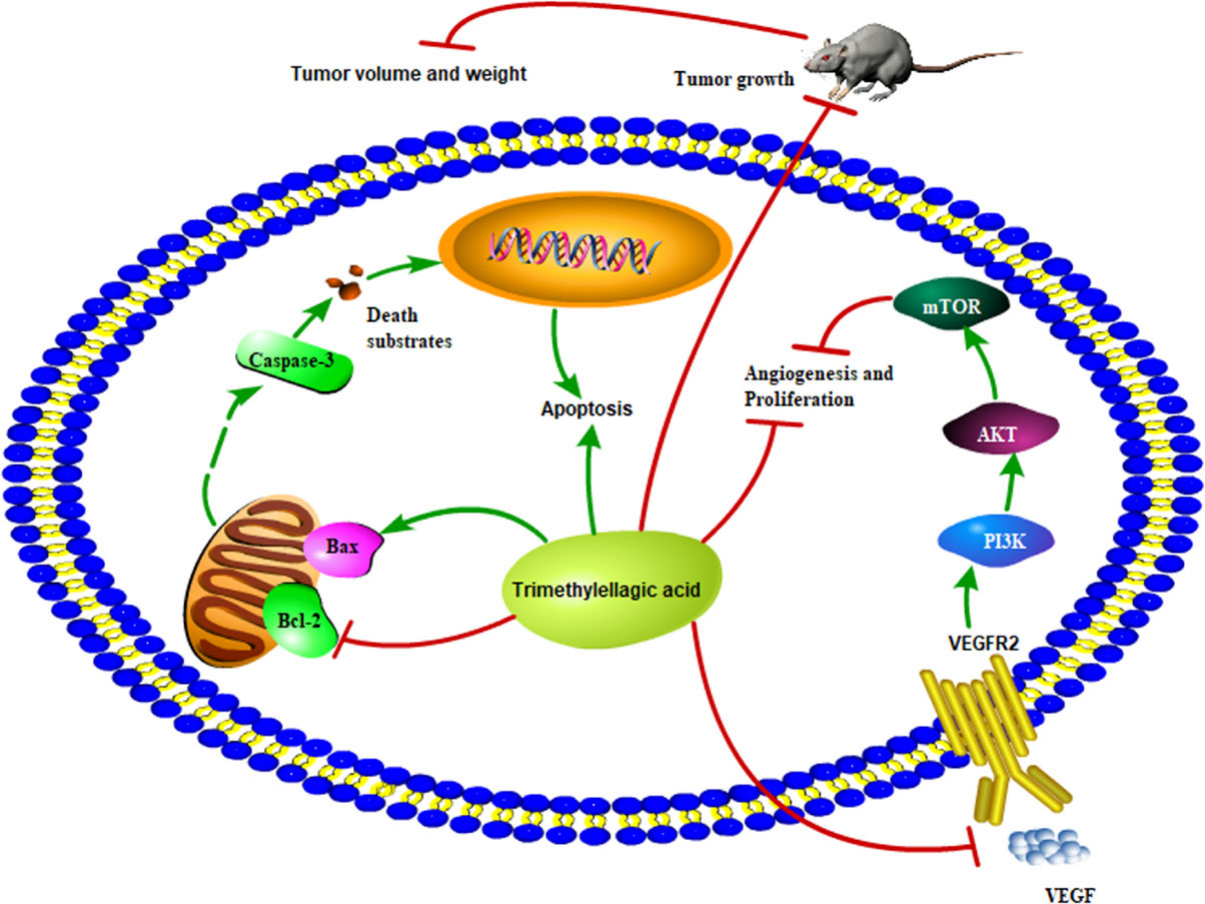
Proposed scheme of the possible mechanism associated with the effects of TMEA on cell growth inhibition and antiangiogenesis.

Chemotherapy and radiotherapy are commonly used for cancer treatment. However, these therapies are associated with severe side effects such as bone marrow inhibition and gastrointestinal toxicity. To overcome these problems, apoptosis has been considered as an important therapeutic target and apoptosis-target drugs have been proven as an effective treatment option for cancers. Apoptosis is programmed cell death that involves the mitochondrial apoptosis pathway and plays a crucial role in the maintenance of tissue homeostasis by regulating cell death (Yao et al., 2017). It is regulated by the Bcl-2 family proteins, which are divided into antiapoptotic proteins, such as Bcl-2, and proapoptotic proteins such as Bax. Reduction of the Bcl-2/Bax ratio is shown to induce the release of cytochrome C from the mitochondria and further activate the mitochondrial-dependent caspase cascade to induce apoptotic cell death (Lan et al., 2010). The present study showed that TMEA significantly inhibited the growth of multiple cancer cells, such as HepG2, A549, and SW620 cells, *via* induction of cellular apoptosis, including activating caspase-3, upregulating Bax, downregulating Bcl-2, and reducing the ratio of Bcl-2/Bax in cancer cells and tumor tissues (**Figure 9**). These findings indicate that the antitumor effect of TMEA is related to apoptosis induction *via* modulation of the apoptotic pathway.

## Conclusion

The present study is the first to demonstrate that TMEA can inhibit the proliferation of various cancer cells *in vitro* and the growth of SW620 tumor xenografts *in vivo* by inducing apoptosis and antiangiogenesis. The anticancer effects of TMEA were associated with effectively targeting the apoptotic and VEGF/PI3K/AKT/mTOR pathways *via* upregulation of Bax, phosphorylation of caspase-3, and downregulation of Bcl-2. Therefore, TMEA may be potentially developed as a novel antitumor agent clinically.

## Data Availability Statement

All data generated or analyzed during this study have been included in this article. Additional information is available on reasonable request to the corresponding authors.

## Ethics Statement

The experimental protocol was approved (Permit No. 20180329) by the Committee on Use and Care of Animals of Southwest Medical University (Luzhou, Sichuan, China).

## Author Contributions

CB, YS, WZ, and JW conceived and designed the experiments. CB, YS, XP, XL, JY, and AW performed the experiments. CB, XP, JY, DQ, and AW analyzed the data. CB, YS, XP, DQ, SC, WZ, JW, and AW wrote the manuscript.

## Funding

This work was supported by Grants from the National Natural Science Foundation of China, Grant number 81774013; the Science and Technology Planning Project of Sichuan Province, China, grant number 18YYJC1177; the Educational Commission of Sichuan Province, China, grant number 18TD0051; and the Science and Technology Program of Luzhou, China, Grant number 2016LZXNYD-T03.

## Conflict of Interest

The authors declare that the research was conducted in the absence of any commercial or financial relationships that could be construed as a potential conflict of interest.

## Abbreviations

5-FU, 5-fluorouracil; AKT, protein kinase B; Asn, asparagine; bFGF, basic fibroblast growth factor; EAC, acetic ether; ECGS, endothelial cell growth supplement; FBS, fetal bovine serum; Gly, glycine; IL-8, interleukin-8; Leu, leucine; mTOR, mammalian target of rapamycin; PDB, protein data bank; PDGFR, platelet-derived growth factor receptor; PE, petroleum ether; PI3K, phosphatidylinositol 3-kinase; TGF-β, transforming growth factor-β; TMEA, 3,3',4'-trimethylellagic acid; VEGF, vascular endothelial growth factor; VEGFR2, vascular endothelial growth factor receptor 2.

## Supplementary Material

Figure S1 The plant sample of *Sanguisorba officinalis* L.

Figure S2 Determination of TMEA HPLC chromatography. **(A)** TMEA dissolved in pure DMSO; **(B)** TMEA stored at -20°C for 7 days.
