# Supplementary figures and images for: Antitumor Effects of Trimethylellagic Acid Isolated From Sanguisorba officinalis L. on Colorectal Cancer via Angiogenesis Inhibition and Apoptosis Induction
Source: Front Pharmacol. 2020 Jan 28;10:1646. doi: 10.3389/fphar.2019.01646 (PMC6997556; doi:10.3389/fphar.2019.01646)

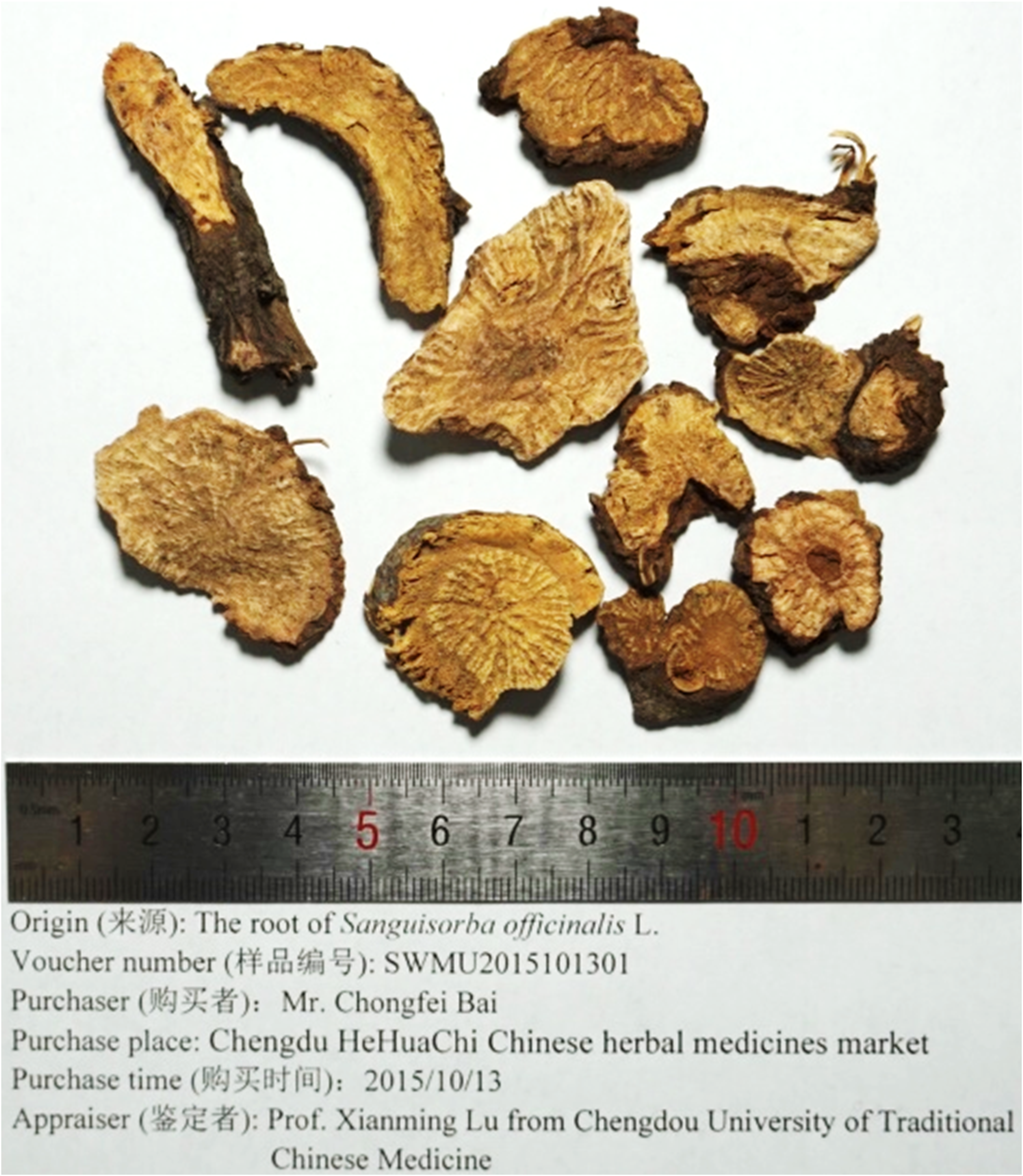

Supplement: Figure S1 — The plant sample of Sanguisorba officinalis L. [file Image_1.tif]

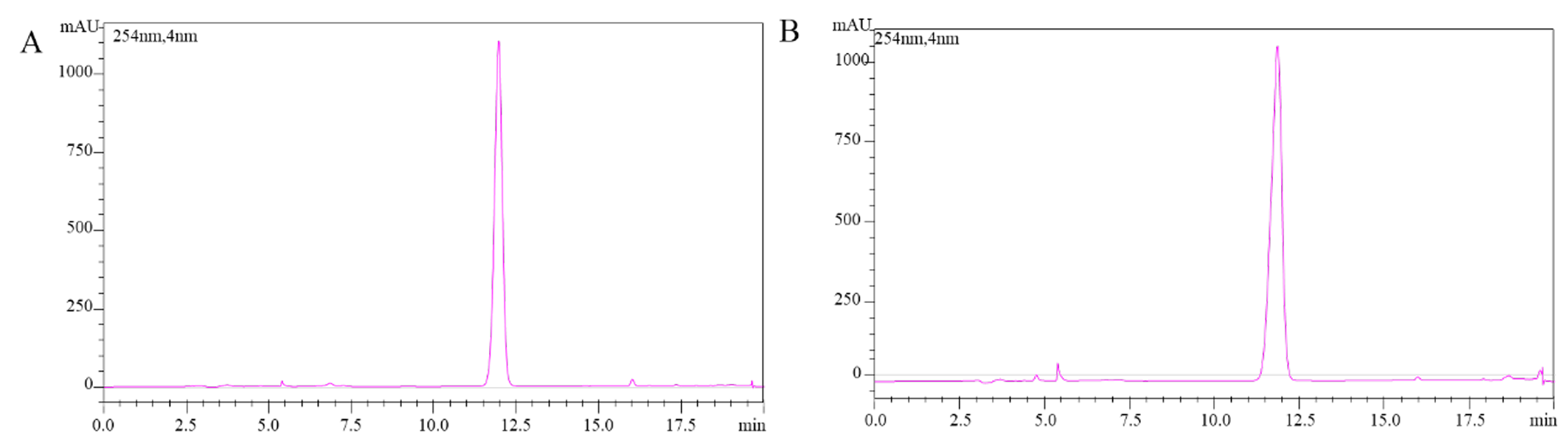

Supplement: Figure S2 — Determination of TMEA HPLC chromatography. (A) TMEA dissolved in pure DMSO; (B) TMEA stored at -20°C for 7 days. [file Image_2.tif]
